# Metabolic Dysfunction-Associated Steatotic Liver Disease Is Characterized by Enhanced Endogenous Cholesterol Synthesis and Impaired Synthesis/Absorption Balance

**DOI:** 10.3390/ijms26157462

**Published:** 2025-08-01

**Authors:** Irena Frankovic, Aleksandra Zeljkovic, Ivana Djuricic, Ana Ninic, Jelena Vekic, Minja Derikonjic, Sanja Erceg, Ratko Tomasevic, Milica Mamic, Milos Mitrovic, Tamara Gojkovic

**Affiliations:** 1 Department of Medical Biochemistry, Faculty of Pharmacy, University of Belgrade, 11000 Belgrade, Serbia; irenafrankovic@yahoo.com (I.F.); ana.ninic@pharmacy.bg.ac.rs (A.N.); jelena.vekic@pharmacy.bg.ac.rs (J.V.); minja.derikonjic@pharmacy.bg.ac.rs (M.D.); sanja.erceg@pharmacy.bg.ac.rs (S.E.); tamara.gojkovic@pharmacy.bg.ac.rs (T.G.); 2 Department of Bromatology, Faculty of Pharmacy, University of Belgrade, 11000 Belgrade, Serbia; ivana.djuricic@pharmacy.bg.ac.rs; 3 Department of Internal Medicine, Faculty of Medicine, University of Belgrade, 11000 Belgrade, Serbia; ratko.tomasevic@med.bg.ac.rs; 4 Department of Gastroenterology and Hepatology, Clinic for Internal Medicine, Clinical Hospital Center Zemun, 11000 Belgrade, Serbia; 5 Department of Laboratory Diagnostics, Clinical Hospital Center Zemun, 11080 Belgrade, Serbia; mamicmilics8@gmail.com; 6 Clinical Department for Gastroenterology and Hepatology, University Medical Center Zvezdara, 11120 Belgrade, Serbia; dr.milosh.mitrovic@gmail.com

**Keywords:** MASLD, non-cholesterol sterols, hepatic steatosis indices, cholesterol homeostasis, endogenous cholesterol synthesis, dietary cholesterol absorption

## Abstract

Cholesterol accumulation plays a significant role in the pathogenesis of metabolic-dysfunction-associated steatotic liver disease (MASLD), yet changes in cholesterol homeostasis in MASLD remain insufficiently investigated. This study aimed to examine alterations in cholesterol synthesis and absorption by measuring plasma levels of endogenous cholesterol precursors (as markers of synthesis) and phytosterols (as indicators of absorption). A total of 124 MASLD patients and 43 healthy individuals were included. Our results showed higher plasma concentrations of lathosterol in the MASLD group (*p* = 0.006), in parallel with comparable concentrations of desmosterol (*p* = 0.472) and all analyzed phytosterols in both groups. Correlation analysis showed that both lathosterol and desmosterol were positively associated with non-invasive hepatic steatosis indices: FLI, HSI, and TyG index (*p* < 0.01, *p* < 0.01, and *p* < 0.05, respectively). Multivariate linear regression further confirmed that these synthesis markers remained significant predictors of FLI (*p* = 0.010), HSI (*p* = 0.013), and TyG index (*p* = 0.002), even after adjusting for other relevant variables. These findings indicate that MASLD is associated with a shift in cholesterol homeostasis towards enhanced endogenous cholesterol synthesis.

## 1. Introduction

The adoption of unhealthy dietary and lifestyle habits over recent decades has contributed to a global rise in obesity and associated health disorders. Among them, metabolic-dysfunction-associated steatotic liver disease (MASLD), being the most prevalent liver disease worldwide [1,2], represents one of the most important challenges for healthcare systems. The contemporary approach to the pathogenesis of MASLD relies on a multiple-hit hypothesis, according to which MASLD development is triggered by obesity-induced dyslipidemia and lipotoxicity, insulin resistance, mitochondrial dysfunction, oxidative stress, and inflammation [3,4]. Accordingly, prevention and treatment of MASLD should focus on each of the metabolic alterations that contribute to its onset and progression.

Dyslipidemia is a key feature of MASLD. Numerous previous studies have highlighted associations between various lipid biomarkers and MASLD [5,6,7,8]. Cholesterol accumulation in hepatocytes, in particular, has been identified as a critical factor in the development of this condition. Cholesterol accumulation in hepatocytes promotes the formation of cholesterol crystals, which in turn activate the activation of the Nod-like receptor pyrin domain containing protein 3 (NLRP3) inflammasome in an NRF2-dependent manner, triggering an inflammatory response [9]. In addition, cholesterol crystals may also deposit in non-parenchymal liver cells, such as Kupffer cells, thereby further contributing to the progression of inflammation and associated pathological processes. Thus, cholesterol lies at the crossroads of dyslipidemia and inflammation, both of which play significant roles in the pathogenesis of MASLD [10]. However, despite the observed elevations in cholesterol levels among MASLD patients, our understanding of cholesterol metabolism in this context remains incomplete. More specifically, the alterations in cholesterol homeostasis during the development of MASLD and their role in exacerbating the condition are not fully elucidated.

Cholesterol homeostasis relies on several key processes: endogenous synthesis, absorption of dietary cholesterol in the intestine, its cellular uptake and efflux, transport, and excretion [10]. The most suitable way to study the balance between cholesterol synthesis and absorption is to determine plasma levels of non-cholesterol sterols (NCSs), which serve as biomarkers of these processes. NCSs are widely recognized as surrogate markers of cholesterol metabolism. Lathosterol and desmosterol are immediate precursors in the Kandutsch–Russell and Bloch pathways of cholesterol biosynthesis, respectively, and are the most commonly used circulating markers for estimating hepatic cholesterol synthesis. In contrast, campesterol, β-sitosterol, and stigmasterol are the most prevalent dietary plant sterols that share the same intestinal absorption route as cholesterol, primarily mediated by the Niemann–Pick C1-like 1 (NPC1L1) transporter, while their efflux back into the intestinal lumen is regulated by ABCG5/G8 transporters. Quantification of these markers enables evaluation of cholesterol absorption efficiency in the small intestine. These NCSs have been validated in both clinical and epidemiological studies as sensitive and specific markers of cholesterol homeostasis [11,12]. Previous studies have shown that in healthy individuals, de novo cholesterol synthesis and intestinal absorption are reciprocally regulated. Thus, an increase in one process is typically accompanied by a decrease in the other [10]. However, this regulatory balance is reportedly disrupted in obesity-related diseases [13,14,15]. Notably, even though hypercholesterolemia is readily observed in MASLD, the concentrations of NCSs in MASLD patients and their relationships have been scarcely explored so far.

In this study, we analyzed differences in NCS levels between patients with MASLD and healthy individuals. Additionally, we explored the associations between NCSs and various clinical and biochemical parameters in MASLD patients, as well as their relationships with scores and indices proposed as markers of the disease.

## 2. Results

Table 1 represents the anthropometric data and basic clinical characteristics of the study participants. There were no significant differences between MASLD patients and the control group in terms of age, gender distribution, smoking, and alcohol consumption, but the proportion of physically active people was lower in the MASLD group. Regarding anthropometric characteristics, BMI and waist-to-hip ratio were higher in the MASLD group. Similarly, this group had higher concentrations of glucose, HbA_1c_, and CRP. ALT activities did not differ between the groups. In contrast, GGT activities were significantly higher in the patient group. Regarding serum lipid parameters, there were no differences in TC and LDL-C levels, but HDL-C concentrations were significantly lower, while TG concentrations were higher in subjects with MASLD compared to controls. Expectedly, hepatic steatosis indices (FLI, HSI, and TyG index) were significantly higher in the MASLD group.

Differences in concentrations of serum NCSs in MASLD patients and control subjects are given in Table 2. The obtained results showed that absolute and relative concentrations of lathosterol were significantly higher in the MASLD group compared to the control group. Desmosterol levels were slightly elevated in MASLD patients, but the difference was not statistically significant. The absolute concentrations of campesterol, stigmasterol, and β-sitosterol did not differ significantly between the groups. However, the lathosterol/β-sitosterol and lathosterol/campesterol ratios were significantly higher in MASLD patients. Although other synthesis-to-absorption sterol ratios did not reach statistical significance, a trend towards increased synthesis was observed in MASLD group. The CSS was significantly higher in MASLD patients, whereas the CAS remained similar between groups. The CSS/CAS ratio was significantly increased in subjects with MASLD. Data distribution and box-plot comparisons for NCSs values and stratification of NCSs concentrations according to BMI values in both groups are given in Supplementary Material S1–S3.

We performed a correlation analysis to examine associations between NCSs and hepatic enzyme activities, parameters of lipid status, and hepatic indices. Spearman’s correlation coefficients are given in Table 3.

Absolute concentrations of desmosterol showed significant positive associations with BMI, albumin, ALT, GGT, TC, LDL-C, TG, FLI, HSI, and TyG index. Lathosterol absolute levels showed similar positive correlations with BMI, ALT, TC, LDL-C, TG, and hepatic indices. On the other hand, campesterol concentrations negatively correlated with BMI and GGT, while they positively correlated with albumin, TC, HDL-C, and LDL-C. The relative concentrations of desmosterol were positively associated with BMI, ALT, and GGT but negatively associated with lipid profile parameters, including TC, HDL-C, and LDL-C. The relative concentrations of lathosterol were positively associated with BMI, ALT, TG, and hepatic indices but negatively associated with HDL-C. In contrast, relative campesterol levels showed negative correlations with BMI, GGT, TG, and TyG index, whereas positive correlations were shown with albumin and HDL. Relative concentrations of stigmasterol and β-sitosterol were inversely associated with albumin, TG, and LDL-C levels.

Composite cholesterol synthesis marker CSS had a significant positive correlation with BMI, ALT, and GGT, lipid parameters TC, LDL-C, and TG, and hepatic indices. CAS was positively associated with TC, HDL-C, and LDL-C, while the CSS/CAS ratio showed consistent positive associations with BMI, ALT, GGT, LDL-C, and TG, as well as with hepatic indices.

Finally, we performed univariate and multivariate linear regression analyses to investigate the independent associations between changes in NCS levels and indicators of MASLD. The MASLD-associated indices, FLI, HSI, and the TyG index, were used as dependent variables in separate analyses. CSS was selected as the most representative marker of disrupted cholesterol balance in MASLD, as significantly increased cholesterol synthesis was observed in this group. The multivariate model was constructed to exclude any parameters already incorporated into the hepatic index calculations while including relevant anthropometric variables (gender and waist-to-hip ratio), albumin as a marker of liver function, HDL-C as an indicator of dyslipidemia, CRP as an inflammatory marker, and HbA_1c_ as a measure of impaired glucose homeostasis. The results are presented in Table 4 and Table 5, respectively. Individual univariate analyses revealed significant independent associations between CSS and all three MASLD-associated indices (Table 4), which remained significant even after CSS was included in the multivariate regression models for each index (Table 5). Corresponding plots for linear regression analyses are given in Supplementary Materials S4 and S5.

## 3. Discussion

In this study, we investigated several markers of cholesterol synthesis and absorption in MASLD. The results obtained suggest that MASLD is associated with alterations in cholesterol homeostasis, which could have a significant impact on disease progression.

The differences in anthropometric and metabolic markers, such as BMI, parameters of glycemia and inflammation, as well as the lipid profile between studied groups (Table 1), indicated typical changes for MASLD in the patient group. MASLD is regarded as a hepatic manifestation of metabolic syndrome, and various etiological factors have been identified as significant contributors to its development, including insulin resistance, altered lipid metabolism with accumulated lipids in the liver, lipotoxicity, cellular inflammation, and oxidative stress [16,17]. Lifestyle habits did not differ between the studied groups. However, we observed a higher percentage of males in the patient group, which is consistent with the higher prevalence of MASLD in men in the general adult population [18].

It should be noted that the term MASLD was only introduced into clinical practice a few years ago [19], when it replaced the previously widely used term non-alcoholic fatty liver disease (NAFLD). As it is a relatively new diagnostic entity, there is a lack of biomarkers or diagnostic scores developed specifically for MASLD. However, multiple studies have investigated whether it is acceptable to fully extrapolate available clinical–laboratory findings of studies on NAFLD to MASLD. It has been shown that the classification of patients according to the former NAFLD and the new MASLD criteria is almost completely consistent [20,21]. Therefore, the non-invasive steatosis indices originally developed for NAFLD should also be applicable to MASLD. Indeed, numerous studies have demonstrated their relevance in the assessment of MASLD [22,23,24,25], although it should be noted that gender differences have been found with regard to the use of steatosis indices in the detection of MASLD [24]. Consistent with this, our results showed significantly higher values for all analyzed steatosis indices in MASLD cases (Table 1). Due to the relatively small sample size, we did not perform a sex-specific analysis in our study, but sex-specific differences should be considered in future studies.

It has been shown that free cholesterol accumulation in liver cells significantly contributes to the pathogenesis of steatosis and MASLD by promoting the formation of cholesterol crystals, which further trigger inflammation, hepatocyte death, and activation of Kupffer cells [9,26]. Therefore, a thorough understanding of cholesterol metabolism in MASLD may be essential for more effective prevention and treatment of this disease. Analysis of NCSs in plasma revealed significant changes in the balance between cholesterol synthesis and absorption in MASLD patients (Table 2). In a study investigating cholesterol metabolism in healthy mice, it was demonstrated that the patterns of deuterium enrichment biosynthetic sterol precursors in plasma closely resemble those observed in the liver but differ from patterns seen in extrahepatic tissues, suggesting that cholesterol homeostasis in the liver is mirrored in the plasma [27]. Higher absolute plasma concentrations of lathosterol in the MASLD group, in parallel with comparable concentrations of desmosterol and all analyzed phytosterols in both groups, indicate that MASLD is associated with a shift in cholesterol homeostasis towards increased endogenous synthesis. Lathosterol, an intermediate of Kandutsch–Russell as well as modified Kandutsch–Russell biosynthetic pathways, is in the bloodstream, and it is a reliable indicator of enhanced cholesterol synthesis [11,27]. Importantly, relative lathosterol concentrations were higher in the MASLD group, suggesting that increased endogenous cholesterol synthesis via the Kandutsch–Russell pathway is a characteristic feature of MASLD, independent of individual TC levels. Previous studies also reported increased de novo cholesterol synthesis in patients with hepatic steatosis [11], which was facilitated by upregulated genetic and epigenetic factors [28]. However, detailed insight into reciprocal relationships between synthesis and absorption processes is still lacking. Interestingly, we found no differences in desmosterol levels between MASLD patients and healthy subjects, although desmosterol, as an intermediate of the Bloch biosynthetic pathway [29], also represents the rate of endogenous cholesterol synthesis. It is noteworthy that several protective effects, such as anti-inflammatory, anti-oxidative, and anti-atherogenic effects, have been attributed to desmosterol [30]. Thus, the relative prevalence of lathosterol over desmosterol in MASLD patients may indicate that the beneficial effects otherwise ascribed to desmosterol are absent. However, it should be emphasized that correlation analyses have shown that both lathosterol and desmosterol are positively correlated with MASLD-associated indices, demonstrating a direct relationship between endogenous cholesterol synthesis and hepatic steatosis.

As mentioned above, the metabolic balance of cholesterol is maintained mainly through the reciprocal regulation of de novo synthesis and intestinal absorption [10]. We found no differences between the study groups in any of the cholesterol absorption markers analyzed (Table 2), despite enhanced cholesterol synthesis being observed in the MASLD group. These results suggest that the increased synthesis is not outweighed by a reduced absorption of dietary cholesterol, which could lead to an overaccumulation of this biomolecule in tissues and cells and, consequently, to lipotoxic effects. To confirm these results, the lathosterol/campesterol, lathosterol/β-sitosterol, and total synthesis/total absorption ratios were higher in the MASLD group. A comparable study was conducted by Simonen et al. [31], wherein the authors demonstrated increased cholesterol synthesis and decreased absorption markers in patients with NAFLD. It should be mentioned that we found a trend towards decreased levels of plant sterols in our MASLD group, but without statistical significance. The lack of significant differences in cholesterol absorption markers’ concentration in our study may be due to the relatively small sample size; therefore, our results should be validated in studies with larger cohorts.

Apart from serving as cholesterol absorption markers, plant sterols also exhibit beneficial metabolic effects in various conditions, including hepatic steatosis [32,33]. Our results (Table 3) confirm these findings, as we observed negative correlations between relative concentrations of campesterol and BMI, activity of GGT, TG levels, and TyG index. In contrast to the cholesterol precursors lathosterol and desmosterol, campesterol showed a positive correlation with HDL-C, thus confirming the previously reported relationship between absorbed phytosterols and HDL particles [34]. In this context, if the balance of cholesterol metabolism is shifted in favor of endogenous synthesis, it could also mean that the beneficial metabolic effects of plant sterols are absent in MASLD cases. Therefore, future efforts should focus on restoring the disturbed cholesterol homeostasis in MASLD patients to ensure favorable health outcomes.

To confirm the observed link between increased de novo cholesterol synthesis and MASLD, we have performed a linear regression analysis (Table 4 and Table 5, respectively). Our results revealed independent associations among cholesterol synthesis markers and MASLD-linked hepatic indices. Importantly, cholesterol synthesis markers retained their significance as modulators of MASLD-related indices, even though our models included variables representing other pathophysiologic processes involved in the development of MASLD. To the best of our knowledge, no independent associations between the cholesterol synthesis rate and MASLD have been explored so far. A recent study based on 2017–2020 NHANES data revealed that the ratio of non-HDL-C to HDL-C is an independent predictor of MASLD [35], thus emphasizing the impact of cholesterol-driven lipotoxicity in MASLD’s development. Considering that endogenous synthesis is one of the main sources of cholesterol in non-HDL particles, the results of our study confirm and extend these findings.

The etiopathogenesis of MASLD is complex, and various factors can contribute to its development. Recent studies imply that gender is an important biological variable in MASLD pathogenesis, as the disease is more prevalent and more severe in males, while estrogen provides protection against it in females [36,37]. The proportion of men was higher in our MASLD group, although a statistical difference was not reached in comparison with the control group (Table 1). Of note, gender was included as a covariate in the multiple linear regression analysis, and our results suggest that the association between cholesterol synthesis and markers of MASLD is independent of gender. It is also known that lifestyle habits can influence the onset and progression of MASLD, with the role of diet and physical activity being particularly emphasized [38]. Our study groups were homogeneous in terms of dietary habits, but the proportion of regularly or occasionally physically active individuals was lower in the MASLD group, confirming the previously reported associations between lower levels of physical activity and the development of MASLD. Also, it should be noted that genetic factors, such as polymorphisms of specific genes involved in maintaining lipid homeostasis, might contribute to MASLD’s onset and severity [39]. Our study did not include these analyses, but future research should focus on the possible synergy between genetic and environmental factors in triggering disturbances of cholesterol balance and, consequently, the occurrence of MASLD.

Apart from synthesis and absorption, cholesterol homeostasis depends on its efflux, conversion to bile acids, and excretion from the body [10]. Recent evidence suggests that cholesterol elimination is also altered in MASLD. It has been shown that cellular cholesterol efflux capacity is decreased in these patients [40]. Also, previous studies of MASLD indicated altered activities of key regulators of reverse cholesterol transport, cellular cholesterol uptake, and its metabolic transformation [41]. In this study, we focused on changes in the two key processes responsible for cholesterol influx, while future research should consider specific markers of cholesterol efflux to fully elucidate this aspect of metabolism associated with MASLD. In line with this, the assessment of mediators and biomarkers of cholesterol efflux, such as ABCG1, ABCA1, LCAT, and CETP, as well as indicators of cholesterol to bile acid conversion, such as CYP7A1, CYP8B1, specific oxysterols as intermediates, and bile acids themselves, should provide comprehensive insight into the changes in cholesterol homeostasis in MASLD.

Limitations of our study include the relatively small sample size, which could prevent certain comparisons from reaching statistical significance. Although the limited sample size is acknowledged as a study limitation, an a priori power analysis was conducted using G*Power (version 3.1.9.7) to estimate the required number of participants based on the observed effect size (Cohen’s d = 0.449). The analysis indicated that a minimum of 126 participants (63 per group) would be needed to achieve 80% power at a significance level of α = 0.05. Furthermore, a post hoc power analysis revealed that with the actual sample size (*n* = 167), the achieved power to detect the observed effect size was 0.81. These findings support the statistical adequacy of the study and help contextualize the limitations related to sample size. Another limitation is that our analyses were limited to markers of endogenous cholesterol synthesis and intestinal cholesterol absorption, while cholesterol efflux and elimination were not considered. Future studies should also include this aspect of cholesterol metabolism. Next, we used hepatic steatosis indices as quantitative indicators for MASLD, although they are primarily derived as biomarkers for NAFLD. However, because NAFLD and MASLD share most clinical and laboratory features, the proposed NAFLD indices are also applicable to MASLD, as confirmed by other studies [20,21,22,23,24,25]. Furthermore, due to the cross-sectional design of our study, it was not possible to investigate a causal relationship between increased cholesterol synthesis and the development of MASLD. Although our results suggest that there is an independent association between elevated cholesterol synthesis markers and indicators of MASLD, it cannot yet be unequivocally confirmed that elevated cholesterol synthesis is a causative factor in the development of MASLD. It should not be neglected that the accumulation of lipids, especially triglycerides, and the resulting damage to hepatocytes in MASLD might be able to trigger an imbalance in liver cholesterol homeostasis. Therefore, it is possible that the observed increase in cholesterol synthesis markers is not the cause but rather the consequence of MASLD, while such deregulation of cholesterol homeostasis could further contribute to disease progression. Future studies employing animal models and cell lines should investigate whether increased de novo cholesterol synthesis precedes MASLD, or vice versa.

In summary, our research results demonstrate the disturbed balance between endogenous cholesterol synthesis via the Kandutsch–Russell pathway and dietary cholesterol absorption in patients with MASLD. Elevated de novo cholesterol synthesis was independently associated with the increase in MASLD-associated hepatic steatosis indices. These findings suggest that measures to maintain adequate metabolic control in MASLD should include efforts to preserve the balance between cholesterol synthesis and absorption. Although causality cannot be confirmed by our study design, a possible involvement of increased endogenous cholesterol synthesis in the development of MASLD offers a new perspective for therapeutic interventions. Mono and combination therapy with lipid-lowering agents has already been shown to improve MASLD-associated indices [42], and our current findings provide an expanded understanding of their effect in MASLD. Statins may be excellent candidates for the therapeutic modulation of increased cholesterol synthesis in MASLD due to their mechanism of action. However, the question of the safety of statins in patients with impaired liver function remains to be answered [43]. Our findings may also have implications for lifestyle interventions implemented as part of the treatment of MASLD patients. Indeed, the observed imbalance between the processes of cholesterol synthesis and absorption could lead to a dietary focus on a limited intake of exogenous cholesterol with a higher intake of plant sterols. Finally, the inclusion of NCSs’ determination in protocols for clinical and laboratory assessment of MASLD could be helpful in stratifying patients and identifying cases that would benefit from specific preventive measures. Overall, further studies are needed to validate our preliminary findings and pave the way for their application in clinical–laboratory practice to progress towards personalized treatment of MASLD.

## 4. Materials and Methods

### 4.1. Patients

This study involved a cohort of 144 adult patients diagnosed with hepatic steatosis confirmed through ultrasound examination. MASLD was diagnosed according to the Delphi consensus criteria [19]. Exclusion criteria comprised viral hepatitis, HIV infection, celiac disease, hereditary liver disorders, and the use of potentially hepatotoxic medications. None of the patients reported excessive regular alcohol consumption (exceeding 140 g/week for women or 210 g/week for men); thus, the diagnosis of metabolic and alcohol-associated liver disease (MAASLD) was excluded. Twenty patients with MASLD who were using statins or other lipid-lowering therapy were excluded, and the final MASLD group comprised 124 subjects. The control group consisted of 43 volunteers who had no ultrasound evidence of hepatic steatosis. Exclusion criteria for the control group comprised the presence of diabetes, coronary heart disease, any other metabolism-associated disorder, and use of lipid-lowering medications.

Patients were recruited between January 2020 and March 2023 at the University Medical Centers Zemun and Zvezdara. Control subjects were also recruited at the University Medical Centers of Zemun and Zvezdara, as well as from the University of Belgrade’s Faculty of Pharmacy.

All participants completed structured questionnaires to collect demographic and clinical information, including age, gender, body weight, height, waist circumference (WC), hip circumference, systolic and diastolic blood pressure, comorbidities, and current medications. Data on lifestyle habits, such as smoking status and alcohol consumption, were also recorded. Based on dietary assessments, all participants adhered to the traditional Central European dietary pattern. Body mass index (BMI) was calculated using the formula weight (kg)/height (m)^2^.

The study protocol was approved by the Ethics Committees of the University of Belgrade–Faculty of Pharmacy and the University Medical Centers Zemun and Zvezdara. The participants were informed of the study’s objectives and gave written informed consent prior to their participation. The study was conducted in accordance with the ethical guidelines outlined in the Declaration of Helsinki.

### 4.2. Laboratory Methods

EDTA plasma and serum were collected after a 12 h fasting period and separated through centrifugation at 1500 rcf for 10 min. Routine laboratory analyses were performed immediately. Concentrations of glucose, albumin, total cholesterol (TC), triglycerides (TG), C-reactive protein (CRP), and HDL-cholesterol (HDL-C) and the liver enzymes alanine aminotransferase (ALT) and gamma-glutamyl transferase (GGT) activities were measured in serum through routine methods using the DxC 700 AU and DxC 480 AU automated analyzers from Beckman Coulter (Brea, CA, USA). Whole blood samples were analyzed for hemoglobin A1c (HbA_1c_) using the immunoturbidimetric method. LDL-cholesterol (LDL-C) was calculated using the Friedewald formula.

Several MASLD-associated indices were used as indicators of hepatic steatosis. Hepatic steatosis index (HSI) values were calculated using the formula 8 × [alanine aminotransferase (ALT)/aspartate aminotransferase (AST)] + body mass index (BMI) + 2 (if diabetic) + 2 (if female) [44]. The fatty liver index (FLI) was calculated using the algorithm based on TG, GGT, BMI, and WC as follows: FLI = (e [0.953 × ln(TG) + 0.139 × BMI + 0.718 × ln(GGT) + 0.053 × WC − 15.745])/(1 + e [0.953 × ln(TG) + 0.139 × BMI + 0.718 × ln(GGT) + 0.053 × WC − 15.745]) × 100 [45]. The triglyceride and glucose index (TyG) was calculated using the formula ln [TG (mg/dL) × glucose (mg/dL)/2] [46].

### 4.3. Non-Cholesterol Sterols’ Determination

Concentrations of NCSs were quantified using gas chromatography with flame ionization detection (GC-FID). Sample preparation and the GC-FID method were performed according to the procedure by Wen-Feng Wu [47] with certain modifications [48]. NCSs were analyzed in serum samples after extraction and derivatization.

Sample handling was performed using borosilicate glassware and high-density plastic labware. Low-density plastics were excluded due to previously demonstrated interference of leached plasticizers with derivatization yield [48]. Glassware was rigorously cleaned following a validated protocol to prevent cross-contamination [48]. Serum samples stored at −80 °C were thawed and mixed just before analysis. Then, 100 μL of sample was transferred into a conical 15 mL glass tube containing 300 μL of internal standard (concentration of 1 mg/mL of 5-α-cholestane dissolved in hexane) previously added and dried in a vacuum concentrator. Then, 1 mL of ethanol (Fisher Chemical^TM^, Landsmeer, The Netherlands) and 960 μL of potassium hydroxide solution (concentration of 8.9 mol/L) were added. The mixture was vortexed for 15 s and heated in a water bath 60 min at 67 °C for saponification. After cooling to room temperature, 1 mL of deionized water and 2 mL of n-hexane were added, briefly vortexed, and centrifuged at 1500 rcf for 5 min. The upper hexane layer was transferred into a glass tube using a glass pipette. The extraction procedure, followed by the addition of 2 mL of n-hexane, was repeated three times. The desalting procedure of the collected n-hexane extract was carried out by adding 4 mL of deionized water to the collected extract, vortexing, and then centrifuging for 5 min at 1500 rcf. The upper layer was transferred into another clean glass tube and dried in a vacuum concentrator for 30 min at 50 °C. After completely drying the extract, 220 μL of derivatization reagent, Sylon^TM^ HTP (Supelco, Bellefonte, PA, USA), was added, and 30 min of incubation in a water bath was performed at 80 °C. After cooling at room temperature, the mixture was dried in a vacuum concentrator. Solid debris was reconstituted in 100 μL of n-hexane before GC analysis.

NCSs’ separation was achieved by using an Agilent HP-5 ((5%–phenyl)-methylsyloxane) non-polar capillary column (30 m × 0.32 mm × 0.25 μm). A splitless liner was used to introduce 1 μL of derivatized extract into the column. The injector and FID detector temperatures were set to 290 °C. A multiramp oven temperature program was used; an initial temperature of 150 °C was held for 3 min, and then it was raised to 250 °C (rate of 20 °C/min), followed by another linear ramp (rate 5 °C/min) until reaching 270 °C, which was held for 30 min. Helium was used as the carrier gas. A constant pressure of 15 psi was applied during the entire runtime of 40.33 min.

The internal standard, which underwent the entire pre-analytical and analytical process alongside the analytes of interest, was used to minimize pre-analytical and analytical variability and, primarily, to calculate extraction yield [48]. Derivatization efficiency was calculated based on the percentage ratio of the peak areas of derivatized and underivatized cholesterol [48].

Chromatographic peaks of desmosterol, lathosterol, campesterol, stigmasterol, and β-sitosterol were identified through comparison with corresponding standards (Supelco, Bellefonte, PA, USA) and quantified according to the standard curves. Calibration curves were constructed using standard concentrations ranging from 1.98 to 31.75 μmol/L for desmosterol, 1.99 to 28.30 μmol/L for lathosterol, 2.52 to 80.60 μmol/L for campesterol, 1.96 to 31.39 μmol/L for stigmasterol, and 3.35 to 80.37 μmol/L for β-sitosterol. Limits of detection (LOD) and quantification (LOQ) for each sterol were calculated based on the signal-to-noise ratio (S/N). As defined during method validation [38], LOD corresponded to an S/N of 3, and LOQ to an S/N of 10. LOD values (μmol/L) for desmosterol, lathosterol, campesterol, stigmasterol, and β-sitosterol were 0.465, 0.401, 0.591, 0.415, and 0.406, while the corresponding LOQ values were 1.209, 1.165, 1.259, 0.744, and 0.914 μmol/L, respectively. Steroid-free serum (MyBioSource, Inc., San Diego, CA, USA) spiked with known concentrations of NCSs was used for quality control procedures. Serum sterol markers were presented as both absolute and relative concentrations. The relative concentrations of sterol markers in serum were calculated by dividing the absolute concentrations of each sterol by the concentration of TC. The cholesterol synthesis sum (CSS) was calculated by summing the absolute concentrations of the sterol synthesis markers desmosterol and lathosterol. The cholesterol absorption sum (CAS) was calculated by summing the absolute concentrations of the sterol absorption markers campesterol, stigmasterol, and β-sitosterol. The CSS/CAS ratio was determined by dividing the sums of absolute concentrations of cholesterol synthesis and absorption markers. Likewise, the ratios of individual markers of cholesterol synthesis and absorption were calculated.

### 4.4. Statistical Analysis

Data distributions were tested using the Kolmogorov–Smirnov test. Normally distributed variables were presented as mean ± standard deviations, and group differences were examined using Student’s *t*-test. Asymmetrically distributed variables were given as medians (interquartile range) and analyzed using the Mann–Whitney U-test. Categorical data were presented as percentages and compared with the Chi-square test. Spearman’s correlation analysis was used to assess associations between tested parameters. Univariate and multivariate linear regression analyses were used to assess independent associations of cholesterol homeostasis markers with MASLD-associated indices. The model for multivariate logistic regression was designed to include various indicators of metabolic disturbances that accompany MASLD while addressing multicollinearity. The variance inflation factor (VIF) and tolerance were used to detect multicollinearity. VIF values for all parameters included in the model were lower than 5, and tolerance values were higher than 0.2. Differences were considered significant if *p* < 0.05. All statistical analyses were executed using the statistical package PASW Statistics 21.0 (IBM, Armonk, New York, NY, USA). A priori and post hoc power analyses were performed using G*Power software (version 3.1.9.7).

## Figures and Tables

**Table 1:** Comparison of anthropometric and clinical characteristics among the studied groups.

Parameter	MASLD (*N* = 124)	Control Group (*N* = 43)	*p*
Age (years)	55.00 (43.00–64.00)	51.00 (41.00–67.00)	0.322
Gender, male (%)	51.6	37.2	0.103
Smoking, yes (%)	33.1	34.2	0.895
Occasional alcohol intake, no (%)	68.4	64.9	0.218
Physical activity, yes (%)	43.0	64.9	0.021
BMI, kg/m^2^	28.96 (26.27–31.22)	25.14 (22.46–26.64)	<0.001
Waist-to-hip ratio *	0.93 ± 0.10	0.83 ± 0.09	<0.001
Systolic pressure (mm Hg) *	127.7 ± 15.4	125.9 ± 16.9	0.331
Diastolic pressure (mm Hg) *	79.9 ± 12.7	78.0 ± 10.6	0.351
Glucose (mmol/L)	5.80 (5.20–6.90)	5.20 (4.80–5.50)	<0.001
HbA_1C_ (mmol/mol)	37.0 (34.4–57.7)	33.0 (30.0–35.0)	<0.001
CRP (mg/L)	3.20 (1.60–5.60)	1.00 (0.50–2.80)	<0.001
Albumin (g/L) *	43.26 ± 4.56	44.52 ± 2.52	0.031
ALT (U//L)	26.5 (17.0–39.0)	23.5 (17.0–28.0)	0.054
GGT (U//L)	29.0 (19.0–44.5)	15.0 (13.0–25.0)	<0.001
TC (mmol/L)	5.20 (4.53–5.91)	5.14 (4.50–5.85)	0.789
LDL-C (mmol/L)	3.10 (2.60–3.90)	3.10 (2.55–3.80)	0.832
HDL-C (mmol/L) *	1.31 ± 0.35	1.70 ± 0.45	<0.001
TG (mmol/L)	1.50 (1.10–2.04)	0.89 (0.71–1.39)	<0.001
FLI	28.56 (1.22–61.85)	4.92 (0.24–28.34)	0.002
HSI	39.14 (33.95–43.56)	32.68 (29.79–34.85)	<0.001
TyG index *	8.95 ± 0.66	8.32 ± 0.48	<0.001

Data are presented as the median (interquartile range) and compared using the Mann–Whitney U test. Categorical data are presented as relative frequencies (%) and compared with the Chi-square test. * Data are presented as the mean ± standard deviation and compared using Student’s *t*-test. FLI—fatty liver index; HSI—hepatic steatosis index; TyG index—trigliceride-glucose index.

**Table 2:** Comparison of serum NCSs among MASLD patients and the control group.

Parameter	MASLD (*N* = 124)	Control Group (*N* = 43)	*p*
Desmosterol (μmol/L) *	6.83 ± 1.70	6.61 ± 1.80	0.472
Lathosterol (μmol/L)	7.12 (4.63–9.55)	5.13 (2.57–8.25)	0.006
Campesterol (μmol/L)	10.94 (7.94–14.14)	11.16 (9.17–14.76)	0.354
Stigmasterol (μmol/L) *	5.14 ± 2.62	4.47 ± 2.04	0.133
β-sitosterol (μmol/L)	16.80 (13.84–24.67)	18.35 (14.80–22.78)	0.770
Desmosterol/cholesterol (mmol/mol) *	1.33 ± 0.31	1.26 ± 0.0.33	0.192
Lathosterol/cholesterol (mmol/mol)	1.38 (0.90–1.82)	0.91 (0.54–1.44)	0.002
Campesterol/cholesterol (mmol/mol)	2.01 (1.56–2.55)	2.25 (1.83–2.73)	0.119
Stigmasterol/cholesterol (mmol/mol)	0.91 (0.68–1.33)	0.77 (0.58–1.17)	0.090
β-sitosterol/cholesterol (mmol/mol)	3.49 (2.71–5.32)	3.49 (2.79–4.49)	0.515
Desmosterol/campesterol	0.65 (0.50–0.87)	0.57 (0.44–0.77)	0.163
Desmosterol/stigmasterol	1.36 (0.96–2.06)	1.42 (1.15–2.39)	0.467
Desmosterol/β-sitosterol	0.39 (0.27–0.52)	0.35 (0.26–0.44)	0.370
Lathosterol/campesterol	0.67 (0.43–1.08)	0.39 (0.21–0.79)	0.001
Lathosterol/stigmasterol	1.39 (0.82–2.40)	1.22 (0.54–2.22)	0.217
Lathosterol/β-sitosterol	0.38 (0.21–0.66)	0.24 (0.12–0.46)	0.026
CSS (μmol/L) *	14.48 ± 5.21	12.23 ± 4.87	0.014
CAS (μmol/L) *	35.78 ± 11.78	36.02 ± 12.09	0.907
CSS/CAS	0.40 (0.29–0.56)	0.32 (0.23–0.47)	0.044

Data are presented as the median (interquartile range) and compared using the Mann–Whitney U test. Categorical data are presented as relative frequencies (%) and compared with the Chi-square test. * Data are presented as the mean ± standard deviation and compared using Student’s *t*-test. CSS—cholesterol synthesis sum; CAS—cholesterol absorption sum.

**Table 3:** Significant correlations of non-cholesterol sterols and sterol markers with other examined parameters.

Parameter	BMI (kg/m^2^)	Albumin (g/L)	ALT (U/L)	GGT (U/L)	TC (mmol/L)	HDL-C (mmol/L)	LDL-C (mmol/L)	TG (mmol/L)	FLI	HSI	TyG Index
Desmosterol (μmol/L)	0.222 **	0.176 *	0.277 **	0.205 **	0.400 **		0.407 **	0.227 **	0.219 **	0.205 **	0.164 *
Lathosterol (μmol/L)	0.260 **		0.195 *		0.297 **		0.355 **	0.324 **	0.236 **	0.290 **	0.294 *
Campesterol (μmol/L)	−0.188 *	0.247 **		−0.170 *	0.529 **	0.417 **	0.461 **				
Desmosterol/cholesterol (mmol/mol)	0.196 *		0.264 **	0.198 *	−0.480 **	−0.248 **	−0.438 **				
Lathosterol/cholesterol (mmol/mol)	0.316 **		0.220 **			−0.269 **		0.274 **	0.194 *	0.320 **	0.260 **
Campesterol/cholesterol (mmol/mol)	−0.297 **	0.223 **		−0.223 **		0.201 *		−0.175 *			−0.172 *
Stigmasterol/cholesterol (mmol/mol)		−0.202 *			−0.324 **		−0.338 **				
β-sitosterol/cholesterol (mmol/mol)		−0.177 *			−0.443 **		−0.447 **		−0.168 *		
CSS (μmol/L)	0.275 **		0.255 **	0.166 *	0.360 **		0.407 **	0.339 **	0.243 **	0.301 **	0.300 **
CAS (μmol/L)					0.245 *	0.234 **	0.198 *				
CSS/CAS	0.205 *		0.246 **	0.174 *			0.203 *	0.195 *	0.221 **	0.221 **	0.182 **

Values represent Spearman’s correlation coefficients. * *p* < 0.05; ** *p* < 0.01. CSS—cholesterol synthesis sum; CAS—cholesterol absorption sum.

**Table 4:** Univariate regression analysis of the associations between CSS and indicators of MASLD.

Dependent variable: FLI				
Parameter	B	S.E.	β	*p*
CSS (μmol/L)	1.313	0.520	0.200	0.013
Dependent variable: HSI				
Parameter	B	S.E.	β	*p*
CSS (μmol/L)	0.319	0.159	0.156	0.047
Dependent variable: TyG index				
Parameter	B	S.E.	β	*p*
CSS (μmol/L)	0.036	0.010	0.270	0.001

**Table 5:** Multivariate regression analysis of the associations between CSS and indicators of MASLD.

Dependent variable: FLI					
Parameter	B	S.E.	β	*p*	Adjusted R^2^
CSS (μmol/L)	1.336	0.506	0.233	0.010	0.464
Gender (m/f)	−9.845	5.764	−0.174	0.092	
Waist-to-hip ratio	86.582	30.230	0.316	0.006	
Albumin (g/L)	−1.657	0.807	−0.209	0.044	
HDL-C (mmol/L)	−12.542	7.209	−0.188	0.087	
CRP (mg/L)	1.822	0.597	0.276	0.003	
HbA_1C_ (mmol/mol)	0.093	0.168	0.056	0.582	
Dependent variable: HSI					
Parameter	B	S.E.	β	*p*	Adjusted R^2^
CSS (μmol/L)	0.317	0.124	0.269	0.013	0.246
Gender (m/f)	0.664	1.408	0.057	0.639	
Waist-to-hip ratio	5.719	7.382	0.101	0.441	
Albumin (g/L)	−0.209	0.197	−0.129	0.292	
HDL-C (mmol/L)	−3.669	1.761	−0.267	0.041	
CRP (mg/L)	0.259	0.146	0.191	0.080	
HbA_1C_ (mmol/mol)	0.056	0.041	0.162	0.179	
Dependent variable: TyG index					
Parameter	B	S.E.	β	*p*	Adjusted R^2^
CSS (μmol/L)	0.041	0.013	0.329	0.002	0.289
Gender (m/f)	0.241	0.146	0.194	0.103	
Waist-to-hip ratio	1.149	0.767	0.190	0.139	
Albumin (g/L)	−0.014	0.020	−0.078	0.507	
HDL (mmol/L)	−0.403	0.183	−0.275	0.031	
CRP (mg/L)	−0.004	0.015	−0.030	0.775	
HbA_1C_ (mmol/mol)	0.010	0.004	0.265	0.026	

All variables are entered as continuous, except gender, which was entered as a categorical variable (1—male).

## Data Availability

The data presented in this study are available upon request from the corresponding author. The data are not publicly available due to privacy and ethical considerations.

## Supplementary Materials

The following supporting information can be downloaded at https://www.mdpi.com/article/10.3390/ijms26157462/s1.
