# Supplementary material for: Metabolic Dysfunction-Associated Steatotic Liver Disease Is Characterized by Enhanced Endogenous Cholesterol Synthesis and Impaired Synthesis/Absorption Balance
Source: Int J Mol Sci. 2025 Aug 1;26(15):7462. doi: 10.3390/ijms26157462 (PMC12347333; doi:10.3390/ijms26157462)
Supplement: Supplementary file 1 [file ijms-26-07462-s001.zip › ijms-3769883 supplementary 1.pdf]

Supplementary material 1. Data distribution for NCSs in MASLD and control group

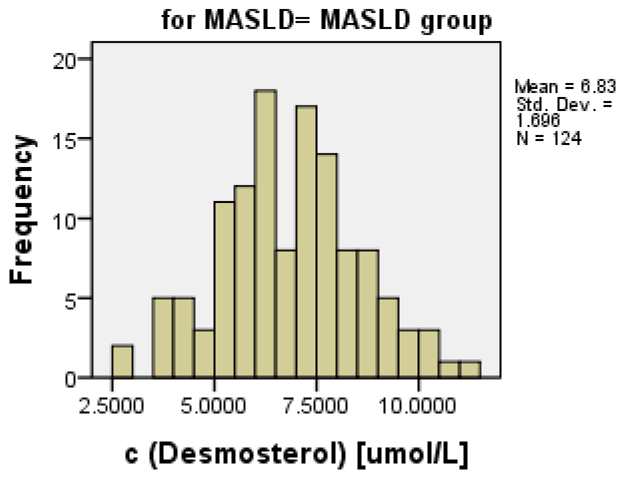

Figure S1.1. Distribution of desmosterol concentrations in the MASLD group

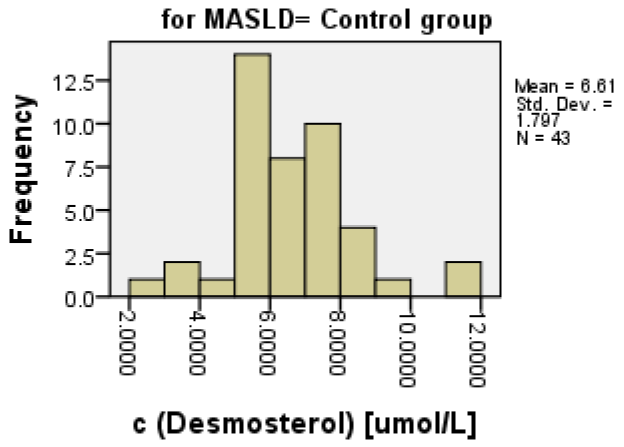

Figure S1.2. Distribution of desmosterol concentrations in the control group

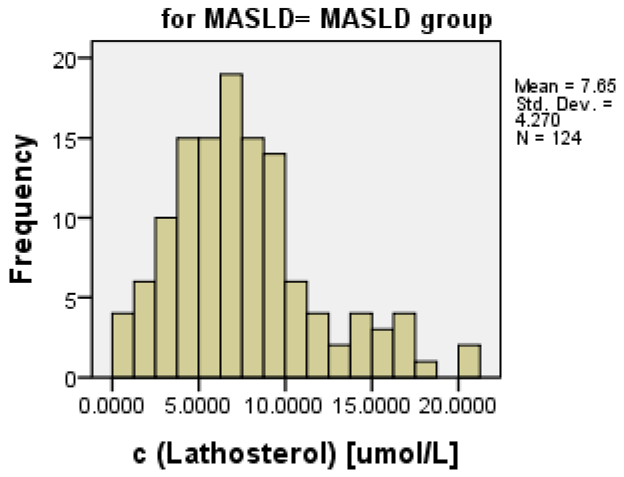

Figure S1.3. Distribution of lathosterol concentrations in the MASLD group

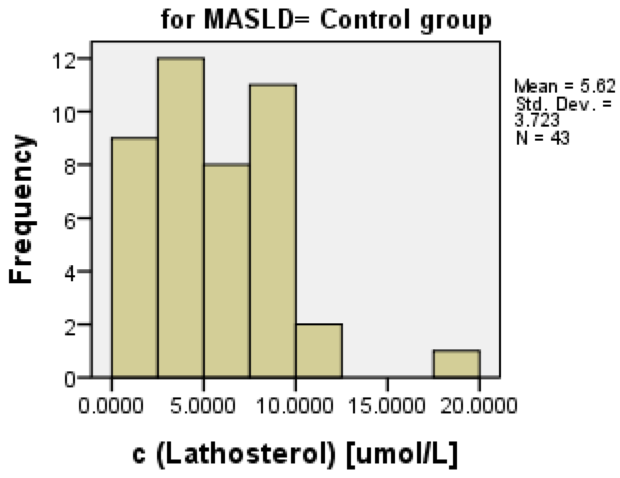

Figure S1.4. Distribution of lathosterol concentrations in the control group

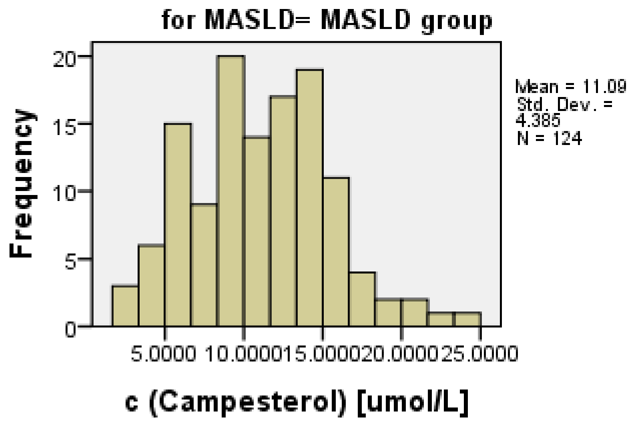

Figure S1.5. Distribution of campesterol concentrations in the MASLD group

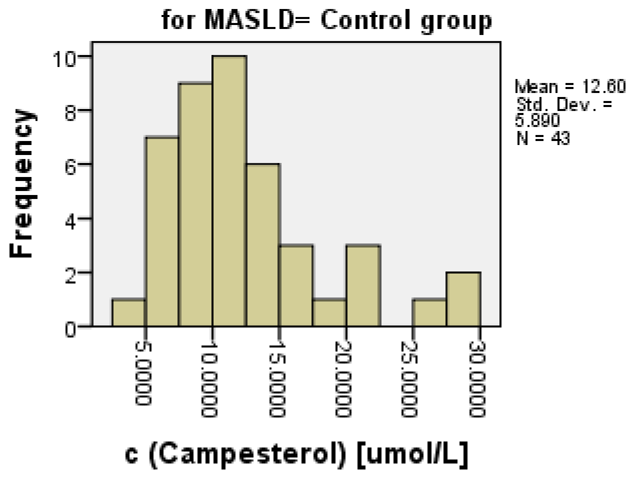

Figure S1.6. Distribution of campesterol concentrations in the control group

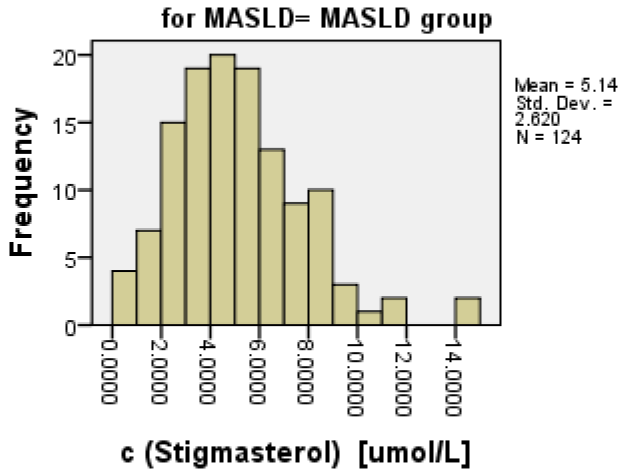

Figure S1.7. Distribution of stigmasterol concentrations in the MASLD group

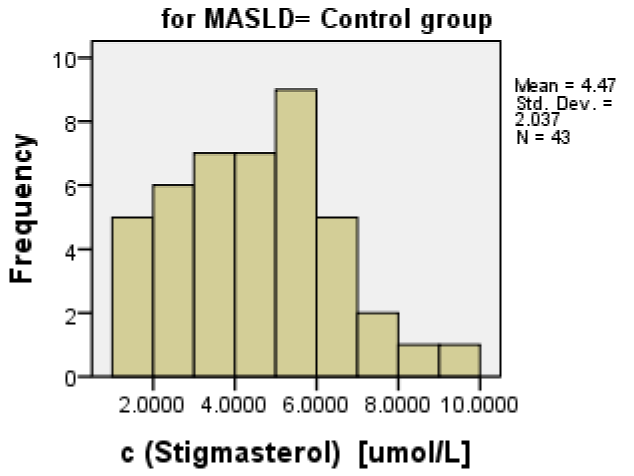

Figure S1.8. Distribution of stigmasterol concentrations in the control group

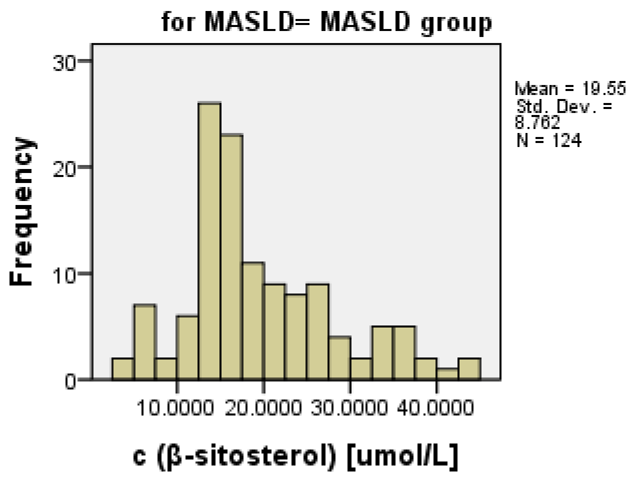

Figure S1.9. Distribution of β-sitosterol concentrations in the MASLD group

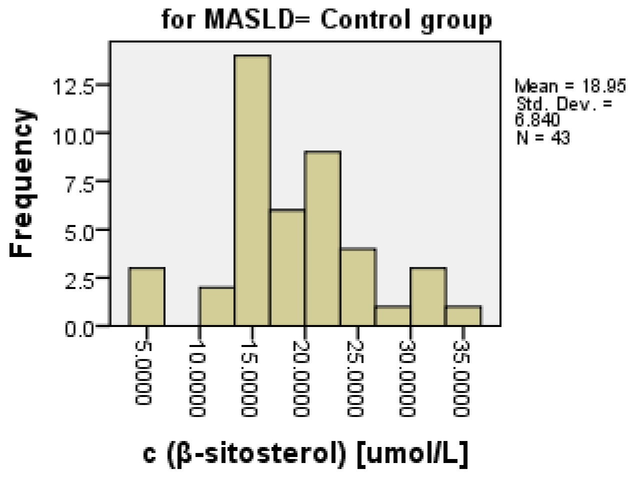

Figure S1.10. Distribution of β-sitosterol concentrations in the control group
