# Supplementary material for: Metabolic Dysfunction-Associated Steatotic Liver Disease Is Characterized by Enhanced Endogenous Cholesterol Synthesis and Impaired Synthesis/Absorption Balance
Source: Int J Mol Sci. 2025 Aug 1;26(15):7462. doi: 10.3390/ijms26157462 (PMC12347333; doi:10.3390/ijms26157462)
Supplement: Supplementary file 1 [file ijms-26-07462-s001.zip › ijms-3769883 supplementary 2.pdf]

Supplementary material 2. Side-by-side box plots for comparison of NCSs concentrations between the analyzed groups

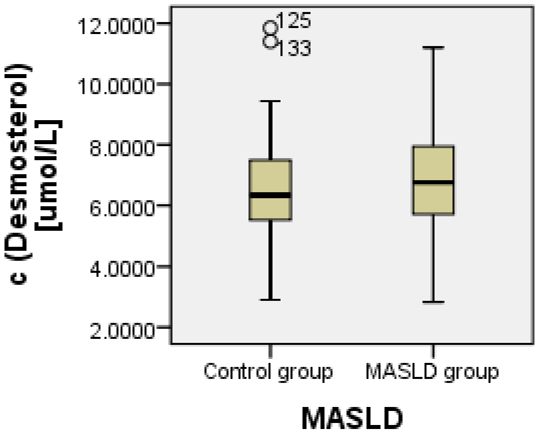

Figure S2.1. Side-by-side boxplots for desmosterol concentrations in the control and MASLD group

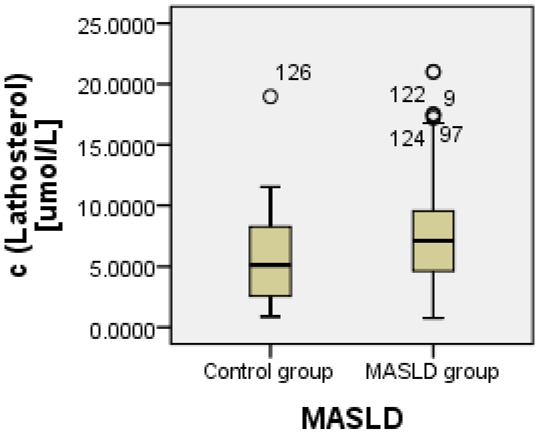

Figure S2.2. Side-by-side box plots for lathosterol concentrations in the control and MASLD group

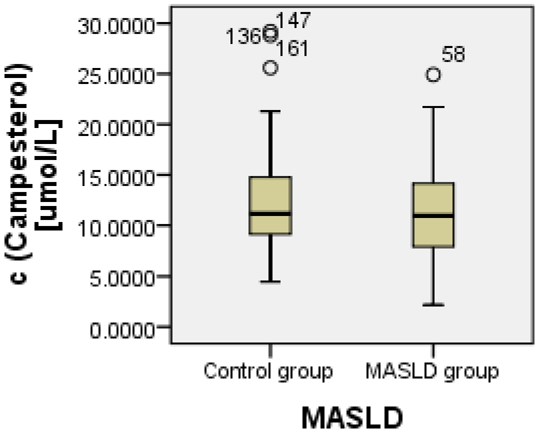

Figure S2.3. Side-by-side box plots for campesterol concentrations in the control and MASLD group

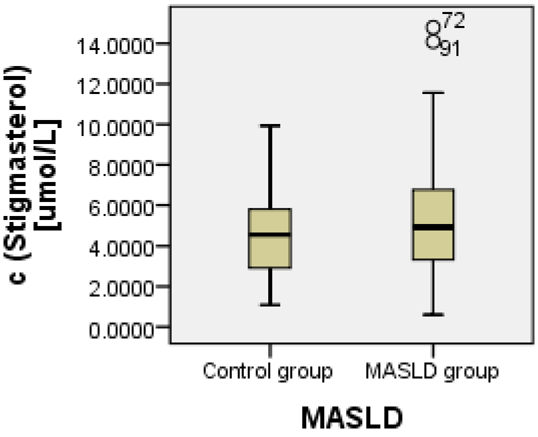

Figure S2.4. Side-by-side box plots for stigmasterol concentrations in the control and MASLD group

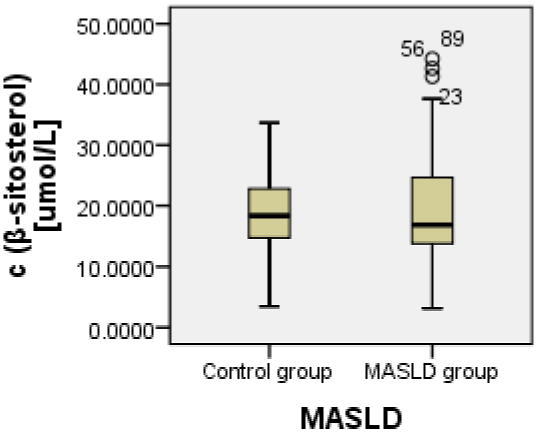

Figure S2.5. Side-by-side box plots for β-sitosterol concentrations in the control and MASLD group
