# Supplementary material for: Metabolic Dysfunction-Associated Steatotic Liver Disease Is Characterized by Enhanced Endogenous Cholesterol Synthesis and Impaired Synthesis/Absorption Balance
Source: Int J Mol Sci. 2025 Aug 1;26(15):7462. doi: 10.3390/ijms26157462 (PMC12347333; doi:10.3390/ijms26157462)
Supplement: Supplementary file 1 [file ijms-26-07462-s001.zip › ijms-3769883 supplementary 3.pdf]

Supplementary material 3. Stratification of NCSs concentrations according to BMI values in the analyzed groups

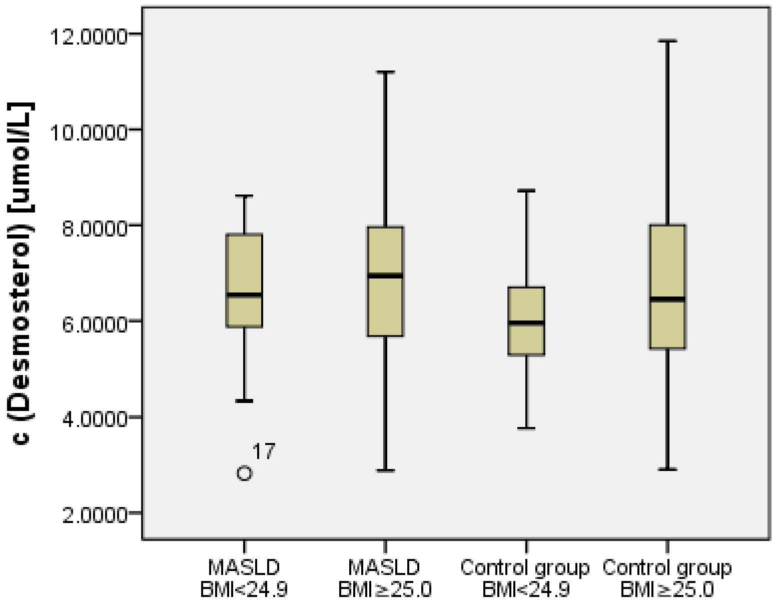

Figure S3.1. Desmosterol concentrations in healthy subjects and MASLD patients with BMI<24.9 kg/m^2 and BMI≥25.0 kg/m^2

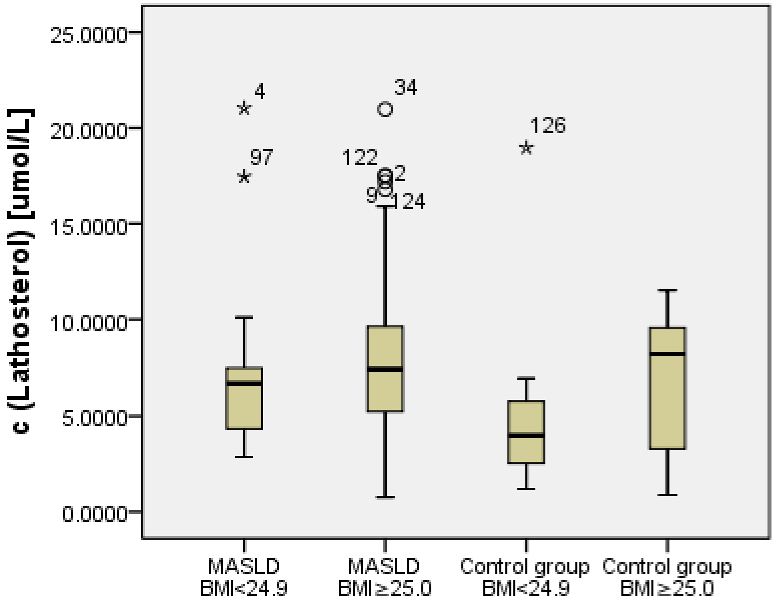

Figure S3.2. Lathosterol concentrations healthy subjects and MASLD patients with BMI<24.9 kg/m^2 and BMI≥25.0 kg/m^2

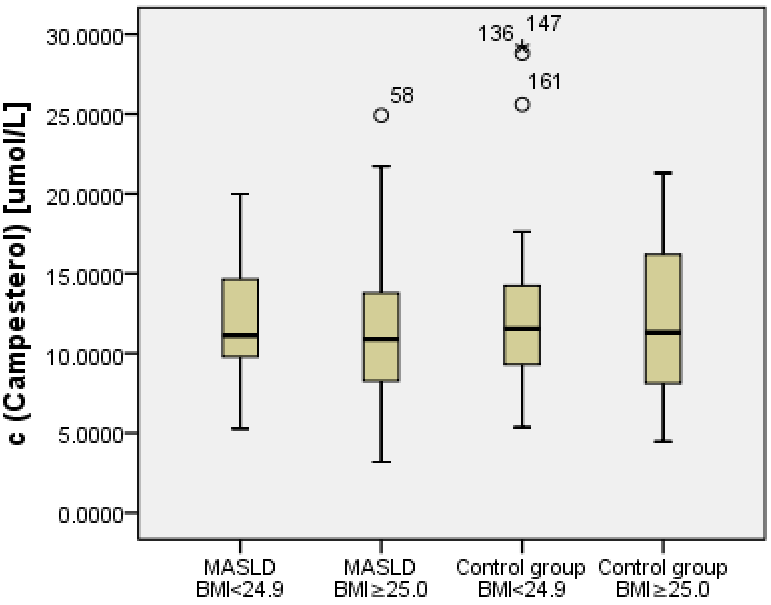

Figure S3.3. Campesterol concentrations healthy subjects and MASLD patients with BMI<24.9 kg/m^2 and BMI≥25.0 kg/m^2

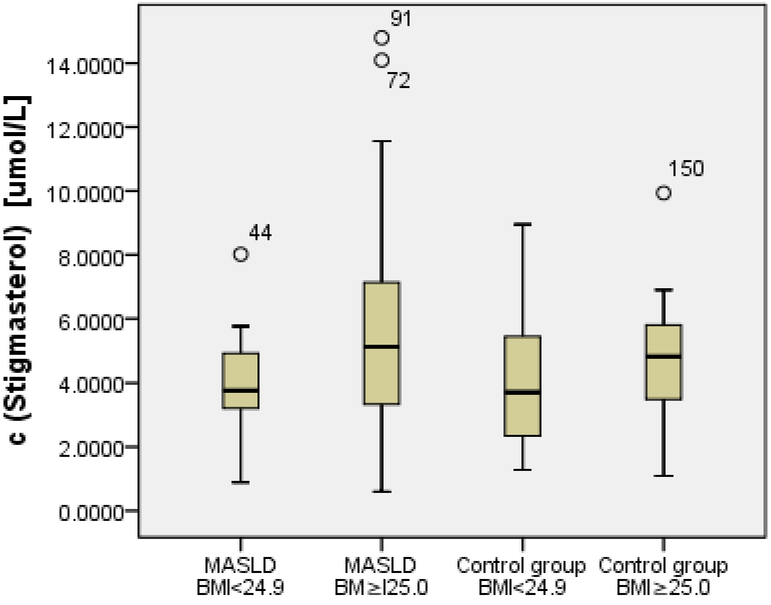

Figure S3.4. Stigmasterol concentrations in healthy subjects and MASLD patients with BMI<24.9 kg/m^2 and BMI≥25.0 kg/m^2

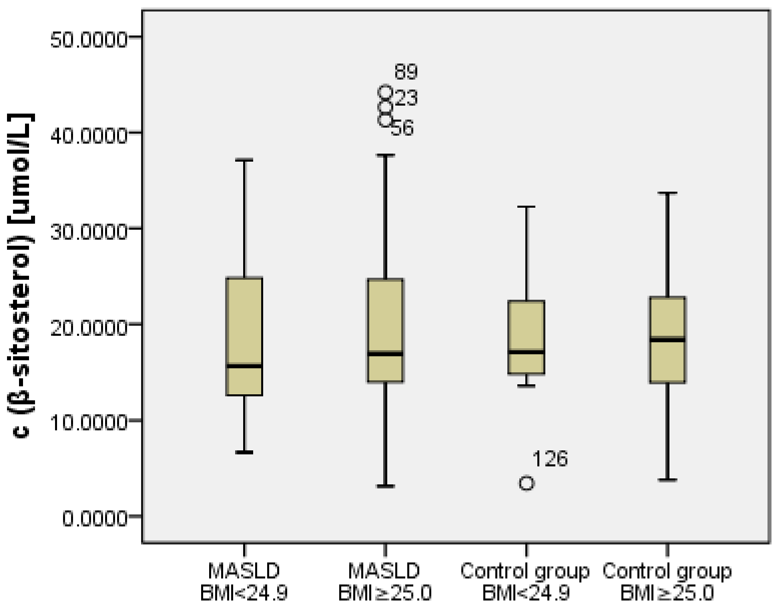

Figure S3.5. β-sitosterol concentrations healthy subjects and MASLD patients with BMI<24.9 kg/m² and BMI≥25.0 kg/m²

Table S3.1. Comparison of desmosterol, latosterol, campesterol, stigmasterol and β-sitosterol in MASLD patients with BMI<24.9 kg/m² and BMI≥25.0 kg/m²

	MASLD BMI<24.9 kg/m²	MASLD BMI≥25.0 kg/m²	
Desmosterol [μmol/L]*	6.48±1.57	6.89±1.70	p=0.380
Lathosterol [μmol/L]	6.70 (4.22-7.68)	7.44 (5.23-9.66)	p=0.323
Campesterol [μmol/L]	11.12 (9.58-15.16)	10.87 (8.17-13.79)	p=0.421
Stigmasterol [μmol/L]*	3.97±1.71	5.41±2.75	p=0.050
β-sitosterol [μmol/L]	15.67 (12.03-25.97)	16.97 (13.97-24.76)	p=0.480

Data presented as median (interquartile range) and compared by Mann-Whitney U test.
*Data presented as mean ± standard deviation and compared by Student t-test

Table S3.2. Comparison of desmosterol, latosterol, campesterol, stigmasterol and β-sitosterol in healthy subjects with BMI<24.9 kg/m^2 and BMI≥25.0 kg/m^2

	Control group BMI<24.9 kg/m^2	Control group BMI≥25.0 kg/m^2	
Desmosterol [μmol/L]*	6.02±1.17	6.87±2.26	p=0.161
Lathosterol [μmol/L]	3.98 (2.49-5.78)	8.24 (3.23-9.57)	p=0.072
Campesterol [μmol/L]	11.53 (9.27-15.08)	11.31 (7.62-16.28)	p=0.762
Stigmasterol [μmol/L]*	4.09±2.19	4.69±2.06	p=0.389
β-sitosterol [μmol/L]	17.13 (14.83-22.84)	18.43 (3.93-22.85)	p=0.965

Data presented as median (interquartile range) and compared by Mann-Whitney U test.
*Data presented as mean ± standard deviation and compared by Student t-test
