# Supplementary material for: Metabolic Dysfunction-Associated Steatotic Liver Disease Is Characterized by Enhanced Endogenous Cholesterol Synthesis and Impaired Synthesis/Absorption Balance
Source: Int J Mol Sci. 2025 Aug 1;26(15):7462. doi: 10.3390/ijms26157462 (PMC12347333; doi:10.3390/ijms26157462)
Supplement: Supplementary file 1 [file ijms-26-07462-s001.zip › ijms-3769883 supplementary 4.pdf]

Supplementary material 4. Univariate linear regression analysis – plots

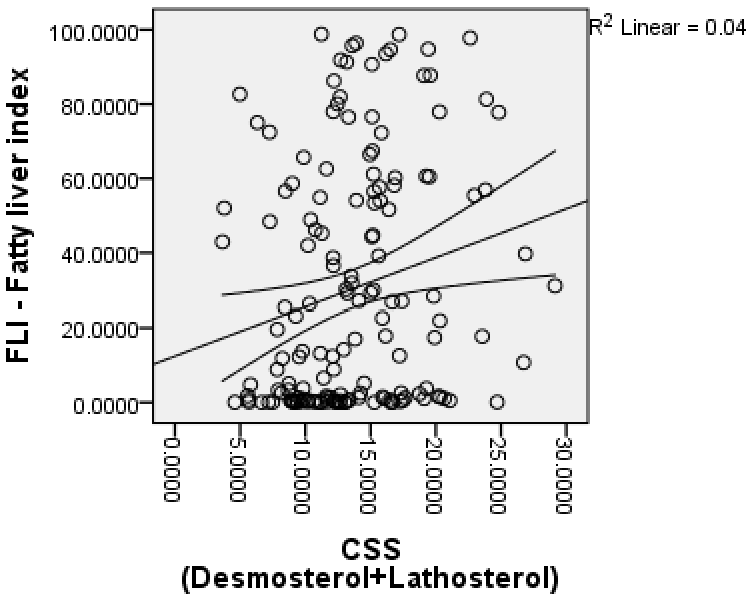

Figure S4.1. Scatterplot for Univariate linear regression model of the association between CSS and FLI

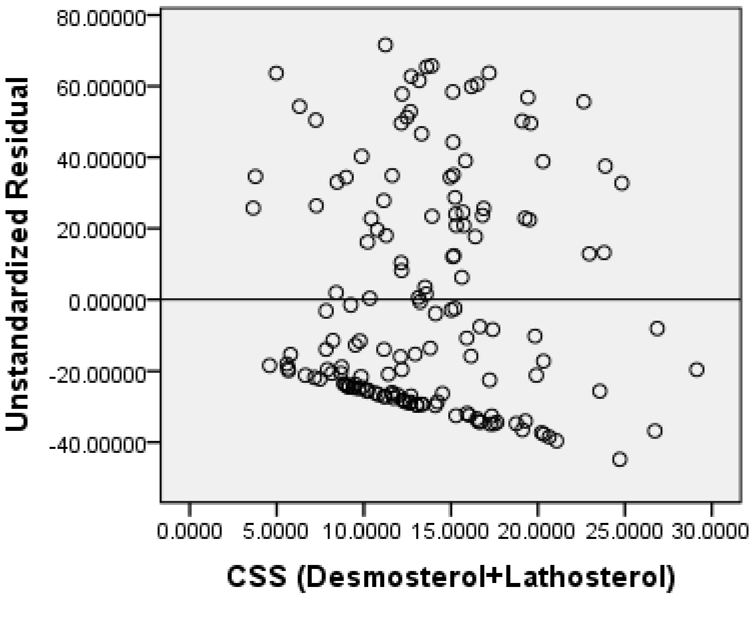

Figure S4.2. Residual plot for Univariate linear regression model of the association between CSS and FLI

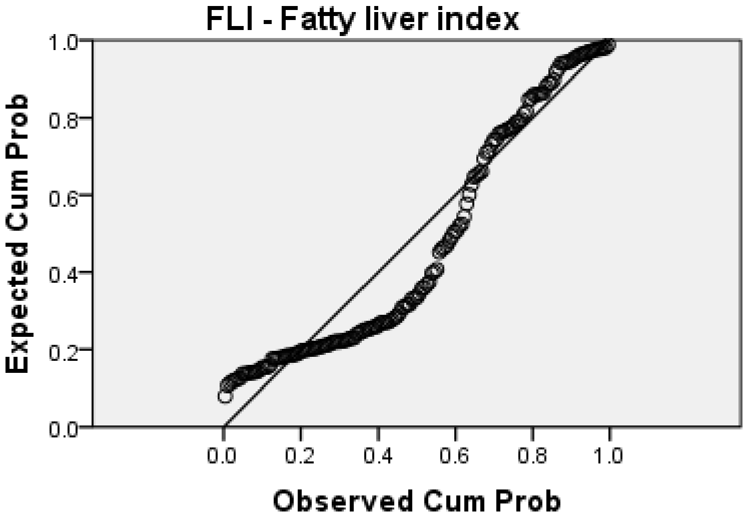

Figure S4.3. Normal P-P Plot of regression standardized residual for Univariate linear regression model of the association between CSS and FLI

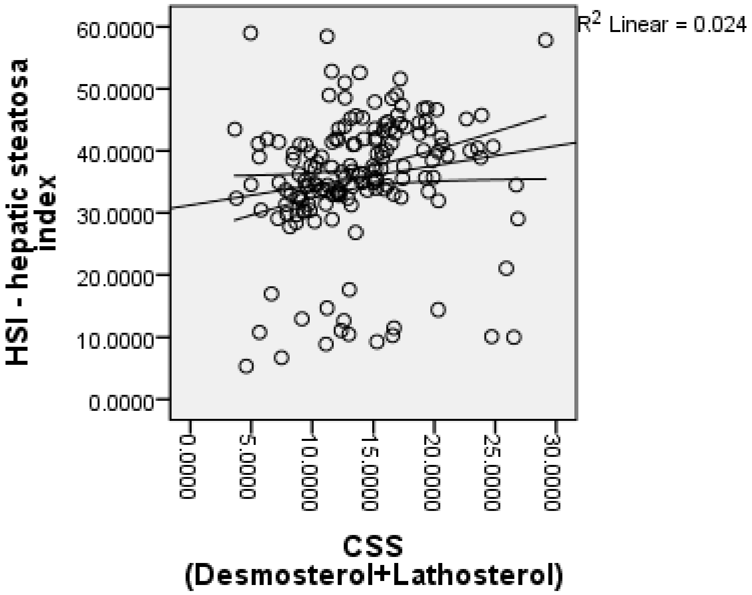

Figure S4.4. Scatterplot for Univariate linear regression model of the association between CSS and HIS

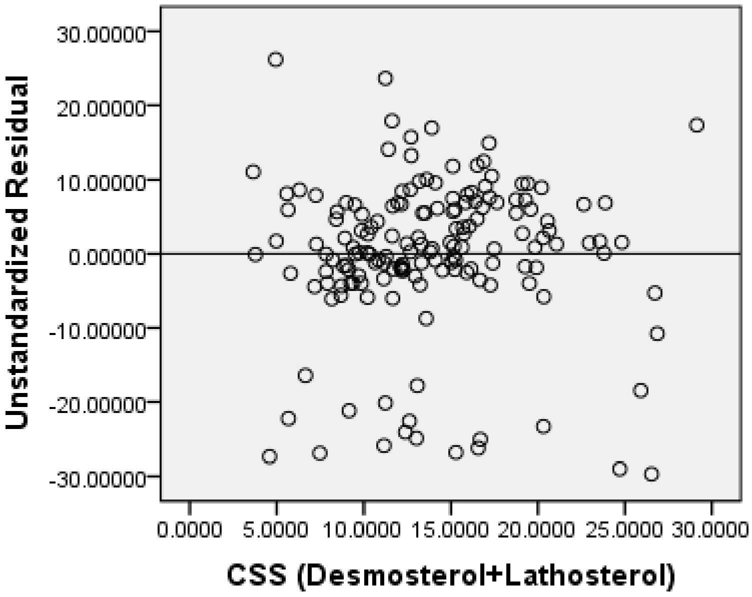

Figure S4.5. Residual plot for Univariate linear regression model of the association between CSS and HIS

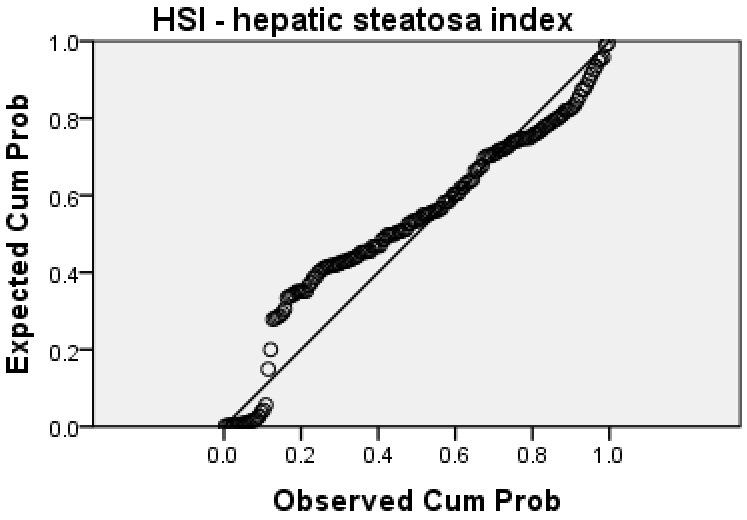

Figure S4.6. Normal P-P Plot of regression standardized residual for Univariate linear regression model of the association between CSS and HIS

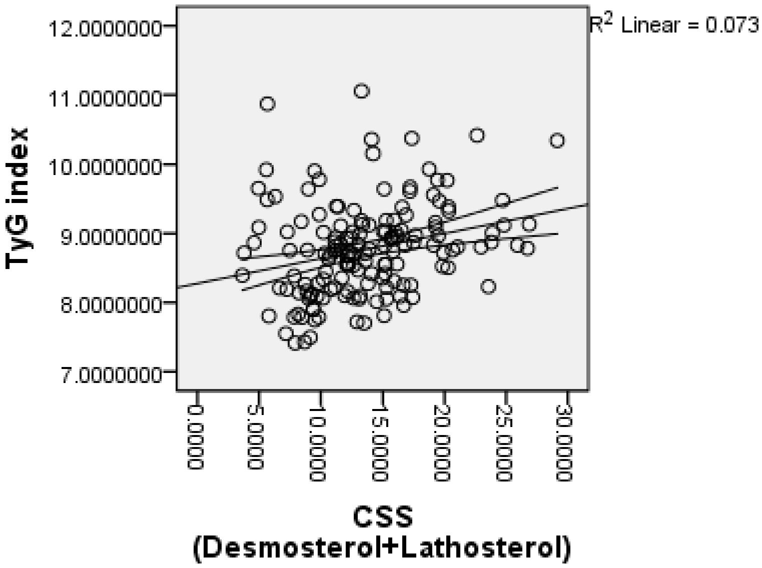

Figure S4.7. Scatterplot for Univariate linear regression model of the association between CSS and TyG index

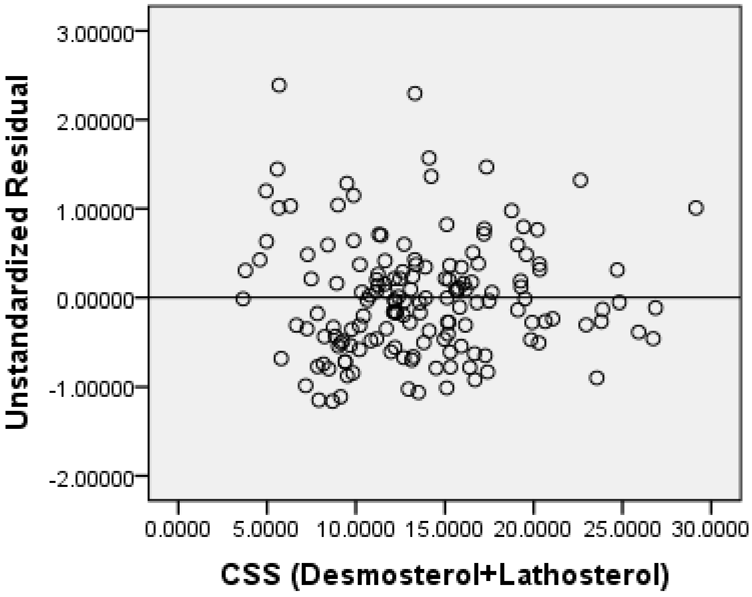

Figure S4.8. Residual plot for Univariate linear regression model of the association between CSS and TyG index

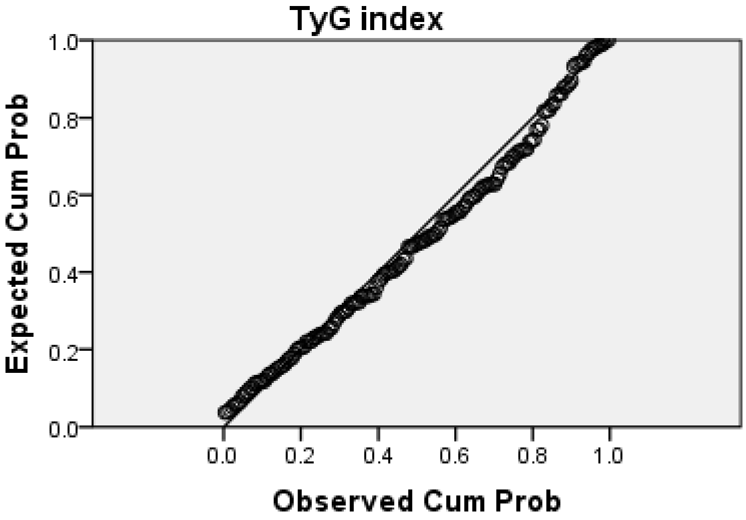

Figure S4.9. Normal P-P Plot of regression standardized residual for Univariate linear regression model of the association between CSS and TyG index
