# Supplementary material for: Metabolic Dysfunction-Associated Steatotic Liver Disease Is Characterized by Enhanced Endogenous Cholesterol Synthesis and Impaired Synthesis/Absorption Balance
Source: Int J Mol Sci. 2025 Aug 1;26(15):7462. doi: 10.3390/ijms26157462 (PMC12347333; doi:10.3390/ijms26157462)
Supplement: Supplementary file 1 [file ijms-26-07462-s001.zip › ijms-3769883 supplementary 5.pdf]

Supplementary material 5. Multivariate linear regression analysis - plots

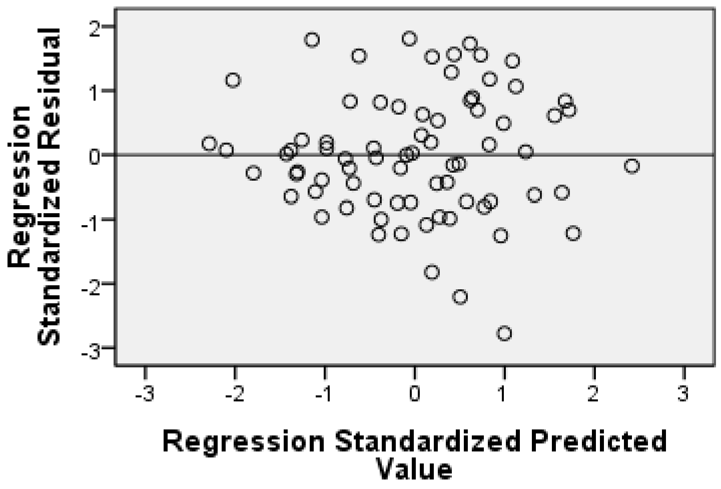

Figure S5.1. Residual plot for Multivariate linear regression model for FLI

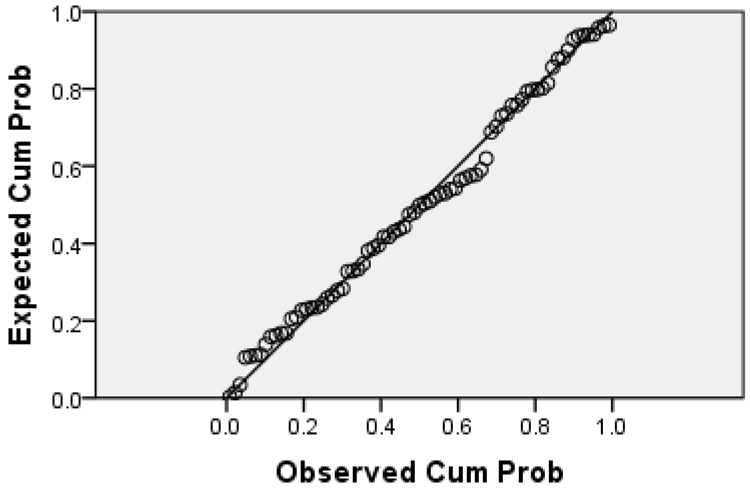

Figure S5.2. Normal P-P Plot of regression standardized residual for Multivariete linear regression model for FLI

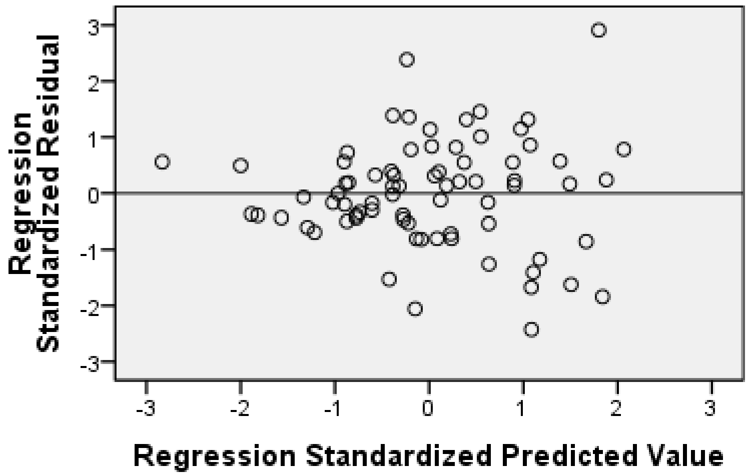

Figure S5.3. Residual plot for Multivariate linear regression model for HIS

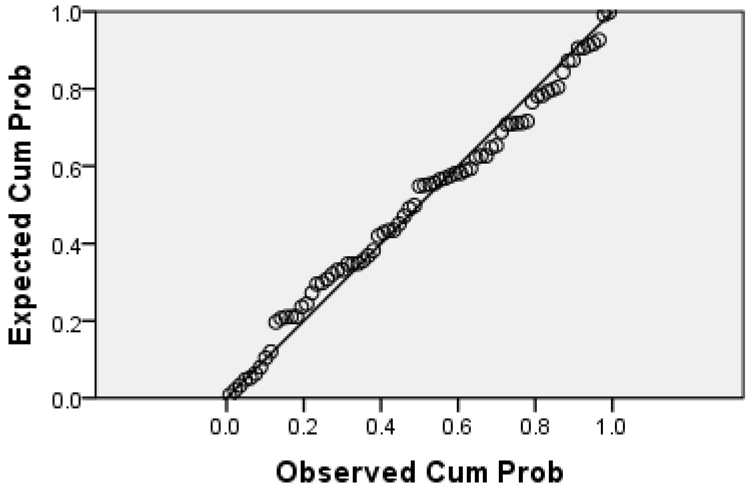

Figure S5.4. Normal P-P Plot of regression standardized residual for Multivariete linear regression model for HSI

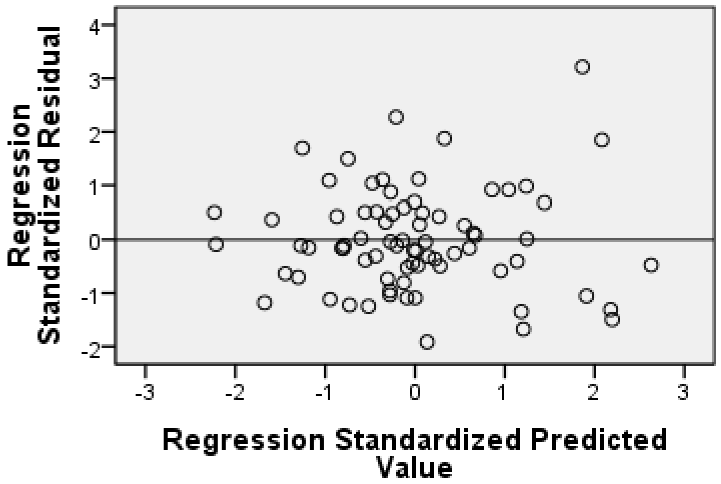

Figure S5.5. Residual plot for Multivariate linear regression model for TyG index

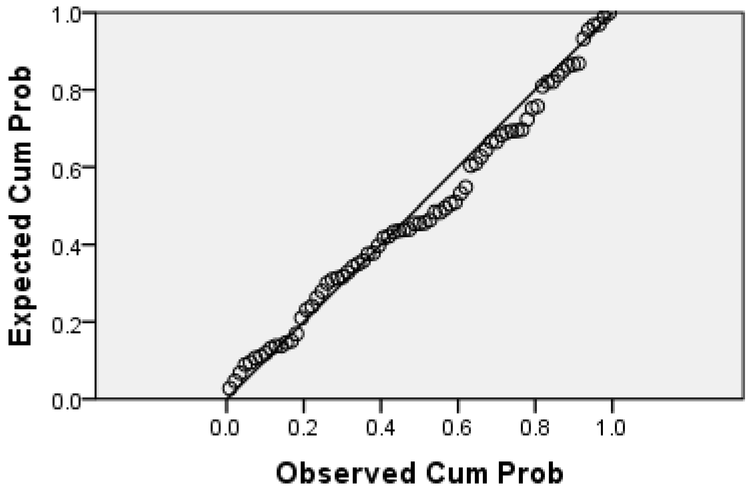

Figure S5.6. Normal P-P Plot of regression standardized residual for Multivariete linear regression model for TyG index
